# Trends in Neuromotor Fitness in 10-to-12-Year-Old Dutch Children: A Comparison Between 2006 and 2015/2017

**DOI:** 10.3389/fpubh.2020.559485

**Published:** 2020-09-25

**Authors:** Manou Anselma, Dorine C. M. Collard, Anniek van Berkum, Jos W. R. Twisk, Mai J. M. Chinapaw, Teatske M. Altenburg

**Affiliations:** ^1^Amsterdam UMC, Vrije Universiteit Amsterdam, Department of Public and Occupational Health, Amsterdam Public Health Research Institute, Amsterdam, Netherlands; ^2^Mulier Instituut, Utrecht, Netherlands; ^3^Amsterdam UMC, Vrije Universiteit Amsterdam, Department of Epidemiology and Biostatistics, Amsterdam Public Health Research Institute, Amsterdam, Netherlands

**Keywords:** physical activity, secular trend, youth, neuromotor fitness, MOPER fitness test

## Abstract

Children with a low level of neuromotor fitness are less skilled to participate in sports activities. Moreover, lower levels of neuromotor fitness are related to adiposity, lower cardiovascular health, and poor self-esteem in children. The aim of this paper was to determine neuromotor fitness in 10–12-year-old Dutch children over a 10-year period. Test scores measured in 2015/2017 (*N* = 533 in 2015, *N* = 941 in 2017) were compared with scores of same-aged children measured in 2006 (*N* = 1986). Neuromotor fitness was assessed using the MOPER fitness test battery, including speed and agility, strength, flexibility, and coordination and upper-limb speed. Data were analyzed using multilevel linear regression models and tobit regression analyses in case of skewed distributions with an excess of zeros. Analyses were stratified by age and gender, and adjusted for level of urbanization. Children in 2015/2017 performed significantly worse on speed and agility (β = 0.8 to 1.1 s), significantly better on coordination/upper-limb speed (β = −1.0 to −0.6 s), and–except for 12-year-old girls–significantly worse on flexibility vs. children in 2006 (β = −3.4 to −1.8 cm). Additionally, upper-body strength was significantly worse among 10-year olds (β = −3.2 to −2.5 s) while leg strength was significantly worse among 11-year-olds in 2015/2017 vs. 2006 (β = −1.8 to −1.7 cm). Trunk strength was worse among 11- and 12-year old boys (β = 1.1 to 1.2 s). In line with a previously observed downward trend in neuromotor fitness among children (1980–2006), we found worse scores on speed and agility, and flexibility in 2015/2017 vs. 2006, stressing the need for interventions aimed at improving neuromotor fitness in order to promote physical activity and future health.

## Introduction

Physical fitness is defined as “a set of attributes that people have or achieve”, which are health- and skill-related attributes that aid performing physical activity (1). Health-related physical fitness consists of aerobic and neuromotor fitness. Neuromotor fitness comprises the components flexibility, coordination, muscle strength, muscle endurance, and speed of movement (1). Better fitness in children is associated with improved future health (2, 3). There is strong evidence for an inverse relationship between neuromotor fitness components and adiposity, cardiovascular health, bone health, and self-esteem (3). Furthermore, children with low levels of neuromotor fitness can experience difficulties with participating in sports activities as they do not have the physical skills required for the complex movements in sports (4). Children who do not participate in sports are also less likely to participate in sports through adolescence and into adulthood (5, 6), thereby increasing the risk for negative health outcomes at all ages (2, 7, 8). Therefore, it is worrisome that worldwide declining trends in childhood physical fitness scores are observed. A systematic review showed a decline in aerobic fitness scores in 6–19-year-old children from 27 countries between 1970 and 2003 (9). Studies on neuromotor fitness showed similar results (10–14). Lower muscular fitness scores in children were observed in different studies on various tests: 10-year-old English children scored lower on handgrip, sit-ups and bent-arm hang in 2008 vs. 1998 (12); Canadian children scored lower on sit-and-reach and handgrip in 2007/2009 vs. 1981 (11); Lithuanian 11–18-year-old children scored lower on standing broad jump, sit-and-reach, and bent-arm hang in 2012 vs. 1992 (14); and Czech children aged 8–9 and 12–13 scored lower on standing broad jump and sit-ups in 2013 vs. 1986 (13). Lower neuromotor fitness scores were also observed on bent-arm hang, sit-and-reach, 10 × 5 meter run, leg-lift, and plate-tapping in a large sample of Dutch 9–12-year-olds in 2006 vs. 1980 (10). However, it is unknown how this trend developed since 2006, as no data on neuromotor fitness of Dutch children have been published since.

The current paper compares neuromotor fitness scores of the same sample measured in 2006 with scores of 10–12-year-old Dutch children measured in 2015 and 2017, to gain insight in neuromotor fitness trends in the Netherlands in the last decade. As the last decade has seen many interventions aiming to improve physical activity of children in the Netherlands (15, 16), the current study can provide insight into the effectiveness of these efforts on neuromotor fitness of children and required future policy directions.

## Materials and Methods

### Recruitment & Participants

For this study, data from three different studies were combined: baseline data collected in 2006 from the “iPlay” -study (10), data from a cross-sectional study in 2015, and baseline data collected in 2017 from the “Kids in Action” project. The iPlay-study included 2,208 children (aged 9–12 years) from 40 primary schools throughout the Netherlands. The original focus of the iPlay-study was to develop and evaluate a program to prevent sport and physical activity related injuries. More details on the study protocol and results are reported elsewhere (10, 17). The cross-sectional study performed in 2015 included 1000 children (aged 9–12 years) from 18 primary schools in and around the city of ‘s Hertogenbosch, the Netherlands. The original focus of this study was to determine the neuromotor fitness levels of children in order to indicate which children had a low fitness level. The Kids in Action study included 656 children (aged 9–12 years) from eight primary schools in and around Amsterdam, the Netherlands. The original focus of Kids in Action was to engage 9–12-year-old children from a low socioeconomic neighborhood in the co-creation of interventions to improve their physical activity and dietary behavior. The eight participating schools were located in neighborhoods of low socioeconomic position (18). In all three studies recruitment of children was conducted via schools. In 2015 and 2017, the MOPER test was conducted as part of a regular physical education class and therefore all children attending the respective physical education class participated in the test. In 2006 all children in grades 7 and 8 were eligible to participate in the study, and those who participated in the study took part in the fitness test. In 2017, four intervention schools were approached and all schools participated. Control schools were contacted (*N* = 22) until four schools agreed to participate (19). Parents of eligible children received a letter containing information on the study and a form to decline their child's participation (passive consent; participation rate 99.1%). In 2015, the MOPER test was conducted as part of the physical education class at 18 primary schools by the “care sport connector” working at the schools. The care sport connector links sports to several other sectors such as care and education. Data from all children participating in those physical education classes were obtained. Because the MOPER test was used as a student monitoring system in school, informed consent was not obtained. In 2006, 520 primary schools were invited to participate in the iPlay-study of which 40 primary schools were included. Parents of eligible children from grades 7 and 8 received a letter containing information on the study and a form to decline their child's participation (passive consent; participation rate: 99.9%). As the three studies only included a small number of children under the age of 10 and over the age of 12 years, these children were not included in the analysis. The Medical Ethics Committee of the VU Medical Center approved the iPlay (2006.129) and Kids in Action (2016.366) study.

### Measurements

#### Procedures

All three studies used the same, standardized protocol of the Motor Performance (MOPER) fitness test (20). The MOPER fitness test consists of eight items in total: 10 × 5 meter run, leg-lifting while laying down, plate-tapping, bent-arm hang, sit-and-reach, arm pull, standing high jump, and a 6-min run test. The MOPER fitness test is considered a valid and reliable measurement tool in children aged 9–18 years (21). The average test-retest coefficient was 0.57 for the high-jump and at least 0.74 for the other items in 9–11-year old boys and girls (21). Assessment of structural validity showed low correlations between test items—correlation coefficients ranging from 0.1 to 0.4—indicating that each item measures a unique characteristic of children's neuromotor fitness (21). In the “Kids in Action” study, the arm-pull test was interchanged for a hand-grip test and in both the “Kids in Action” and “iPlay” study, the 6-min run test was not conducted for practical reasons. Therefore, these tests were not included in the analysis. Table 1 presents a description of the included items of the MOPER fitness test and their metric units.

**Table 1:** Description of the MOPER fitness test items used in this study.

	**Item**	**Characteristic**	**Description**	**Unit**
1.	10 × 5 meter run	Speed and agility	10 times running between 2 lines with a five meters distance as fast as possible, 2 attempts	s
2.	Plate-tapping with one hand	Coordination and upper limb speed	Tapping two plates alternately with the dominant hand 50 times as fast as possible, 2 attempts	s
3.	Bent-arm hang	Upper body strength	Hanging from a horizontal bar with bended arms as long as possible, 1 attempt	s
4.	Standing high jump	Explosive leg strength	Jumping up from a standing position as high as possible, 2 attempts	cm
5.	Leg-lifting while laying down	Trunk and leg strength	Lifting outstretched legs 10 times while laying on back as fast as possible, 1 attempt	s
6.	Sit-and-reach	Flexibility	Reaching from sitting position with outstretched legs and arms as far as possible, 3 attempts	cm

The MOPER test was conducted during a physical education lesson in the school's gym. In 2006 the MOPER was conducted at the beginning of the school year (September), in 2015 and 2017 at the end of the school year (April-May). The children had no previous experience with the test and were not familiarized with the test items. Trained researchers, physical education teachers and/or sports instructors conducted the tests. The participating children were divided into groups of two to four children and they performed the test items one by one. The participants performed the MOPER test bare foot and were encouraged by the instructors to perform optimally.

#### Covariates

Children's age and gender were obtained prior to the MOPER test. Urbanization was included as a confounder as it has been associated with neuromotor fitness in children (22). Data on the degree of urbanization of the school's neighborhood was obtained from Statistics Netherlands (23). The classification of population density in a neighborhood was divided into five categories: extremely high density (>2,500 addresses per km^2^), high density (1,500–2,000 addresses per km^2^), moderate density (1,000–1,500 addresses per km^2^), low density (500–1,000 addresses per km^2^), and extremely low density (<500 addresses per km^2^).

#### Statistical Analyses

Descriptive characteristics and fitness test data were analyzed using means, standard deviations or medians and 25^th^-75^th^ percentiles, and frequencies. For the fitness data first assumptions of normality, linearity, homoscedasticity and the absence of multicollinearity were checked.

The data collected in 2015 and 2017 were combined to increase the sample size. The comparison between the 2006 and 2015/2017 fitness test scores was made using multilevel linear regression analyses for normally distributed variables. The multilevel analyses accounted for the hierarchical structure of the data by adjusting for the clustering of children's test results within schools (24), e.g., due to different physical activity programs or culture between schools. The residuals of two tests were not normally distributed. The “leg-lifting” scores were log-transformed resulting in a normal distribution of the residuals, and therefore multilevel linear regression analyses were used. Due to the skewed distribution and excess of zeros, differences in “bent-arm hang” were analyzed using tobit regression analysis. All analyses were stratified by age and gender, to provide insight in possible age- and gender-specific trends. Analyses were adjusted for the degree of urbanization of the school's neighborhood. Betas (β) and 95% confidence intervals (CI) are reported; the betas of the log-transformed data have to be interpreted as a ratio. *Post-hoc* power calculations showed that the sample size is sufficient to detect a difference of 3-8% as statistically significant. The statistical analyses were conducted using IBM SPSS Statistics 22.0.

## Results

Children were on average 10.8 (SD = 0.7) years old (51% girls) in 2006 and 11.0 (SD = 0.8) years old (52% girls) in 2015/2017. In the 2006 sample, 41% of children attended a school in a high or extremely high density neighborhood, while this was 62% in the 2015/2017 sample.

Table 2 (girls) and Table 3 (boys) provide the results of all fitness tests (mean and standard deviation, median and interquartile range, beta and confidence interval), stratified by age. Both girls and boys in all age categories performed significantly worse on the “10 × 5 meter run” in 2015/2017 vs. 2006 and significantly better on “plate-tapping”. Girls and boys in 2015/2017 ran about 1 s (β ranging from 0.8 to 1.1 s, depending on age and gender) slower than girls and boys in 2006, and were almost a second (β ranging from −1.0 to −0.6 s) faster on “plate-tapping”. “Sit-and-reach” scores were significantly worse in 2015/2017 vs. 2006 in girls (β ranging from −3.4 to −1.8 cm) and boys (β ranging from −2.8 to −2.3 cm), except for 12-year-old girls. Girls and boys in all age categories performed worse in 2015/2017 vs. 2006 on the “bent-arm hang” test, but this was only significant in 10-year-old girls (β = −3.2 s) and boys (β = −2.5 s). 11-year-old girls (β = −1.8 cm) and boys (β = −1.7 cm) scored significantly worse on “standing high jump” in 2015/2017 vs. 2006.

**Table 2:** MOPER fitness test scores for girls in 2006 and 2015/2017, stratified by age.

**Girls**	**10-year-olds**			**11-year-olds**			**12-year-olds**		
	**2006**	**2015/2017**	**%**	**2006**	**2015/2017**	**%**	**2006**	**2015/2017**	**%**
10x5 meter run (s)^1^ x̄ (SD)	20.1 (1.5)	21.2 (1.8)	+4.9	19.9 (1.5)	20.9 (2.0)	+5.0	19.8 (1.6)	20.7 (2.1)	+4.5
β (95% CI)^2^ *n*	1.0 (0.6; 1.4)^***^ 372	222		0.9 (0.6; 1.3)^***^ 516	329		0.8 (0.3; 1.4)^**^ 109	213	
Bent-arm hang (s)^3^^,^ ^4^ *x~* (IQR)	6.0 (3.0–14.0)	5.0 (1.0–10.0)	−16.7	6.0 (2.0–13.0)	4.0 (1.0–10.0)	−33.3	6.0 (2.0–12.0)	4.0 (1.0–11.0)	−33.3
β (95% CI)^2^ *n*	**−3.2 (−5.2;** −1.1)^**^ 372	224		−0.8 (−2.4; 0.7) 521	330		−0.8 (−3.3; 1.6) 109	213	
High jump (cm)^3^ x̄ (SD)	35.8 (6.3)	34.6 (6.4)	−3.4	37.3 (6.3)	35.2 (6.5)	−5.6	37.5 (6.4)	36.4 (6.3)	−2.9
β (95% CI)^2^ *n*	−0.6 (−2.2; 0.9) 373	224		–1.8 (–2.9; –0.8)^**^ 520	330		−1.4 (−3.0; 0.7) 109	213	
Leg-lifting (s) ^1^^,^ ^4^^,^ ^5^ *x~* (IQR)	16.3 (13.9–19.1)	14.8 (13.2–17.6)	−9.2	16.5 (14.2–19.9)	15.9 (13.9–20.0)	−3.6	16.3 (13.8–21.0)	15.4 (13.3–18.3)	−5.5
β (95% CI)^2^ *n*	1.0 (0.9–1.1) 369	223		1.0 (1.0–1.1) 519	330		1.0 (0.9–1.1) 107	212	
Sit-and-reach (cm)^3^ x̄ (SD)	30.4 (6.0)	28.4 (7.1)	−6.6	30.2 (6.2)	26.8 (7.3)	−11.3	29.1 (6.6)	28.1 (7.7)	−3.4
β (95% CI)^2^ *n*	–1.8 (–3.2; –0.4)^*^ 373	224		–3.4 (–4.4; –2.4)^***^ 515	330		−1.1 (−3.0; 0.7) 109	213	
Plate-tapping (s) ^1^ x̄ (SD)	15.2 (1.8)	14.6 (2.0)	−3.9	14.4 (1.8)	13.7 (2.0)	−4.9	14.1 (1.7)	13.2 (1.8)	−6.4
β (95% CI)^2^ *n*	–0.8 (–1.3; –0.3)^**^ 372	224		–0.7 (–1.1; –0.4)^***^ 521	330		–0.9 (–1.4; –0.3)^**^ 109	213	

1 A lower value indicates a better test score

2 2015/2017 vs. 2006

3 A higher value indicates a better test score

4 Data not normally distributed, therefore the median (x~) and interquartile range (IQR) (25^th^-75^th^ percentiles) are provided

5 Data have been log-transformed, therefore the β should be interpreted as a ratio. Bold is significant, by

* p < 0.05,

** p < 0.01,

*** *p < 0.001*.

**Table 3:** MOPER fitness scores for boys in 2006 and 2015/2017, stratified by age.

**Boys**	**10-year-olds**			**11-year-olds**			**12-year-olds**		
	**2006**	**2015/2017**	**%**	**2006**	**2015/2017**	**%**	**2006**	**2015/2017**	**%**
10 × 5 meter run (s)^1^ x̄ (SD)	19.7 (1.5)	20.7 (2.2)	+5.1	19.4 (1.6)	20.4 (2.2)	+5.1	19.2 (1.5)	20.1 (2.0)	+4.7
β (95% CI)^2^ *n*	0.9 (0.4; 1.4)^**^ 366	205		1.0 (0.5; 1.5)^***^ 459	298		1.1 (0.5; 1.6)^***^ 153	204	
Bent-arm hang (s)^3^^,^ ^4^	9.0 (4.0–20.0)	5.0 (1.0–10.0)	−44.4	9.0 (4.0–18.0)	7.0 (2.0–16.0)	−22.2	10.0 (4.0–21.0)	7.0 (1.0–15.8)	−30.0
*x~* (IQR) β (95% CI)^2^ *n*	**−2.5 (−5.0;** −0.0)^*^ 365	205		–1.5 (–3.5; 0.6) 458	298		–2.9 (–5.9; 0.1) 153	204	
High jump (cm)^3^ x̄ (SD)	36.8(5.8)	35.8(6.3)	−2.7	38.6(6.5)	36.8(6.7)	−4.7	39.6(6.5)	39.2(7.0)	–1.0
β (95% CI)^2^ *n*	–0.8 (–2.2; 0.5) 367	205		**−1.7 (−3.0;** −0.4)^**^ 460	298		–0.5 (–2.1; 1.2) 153	204	
Leg-lifting (s)^1^^,^ 4^,^ ^5^ *x~* (IQR)	16.4 (14.2–20.0)	15.8 (13.4–19.8)	–3.7	16.7 (13.9–21.4)	16.8 (13.9–21.0)	+0.6	16.4 (13.9–20.8)	17.2 (13.5–22.3)	+4.9
β (95% CI)^2^ *n*	1.0 (0.9–1.1) 363	205		1.1 (1.0-1.2)^*^ 449	298		1.2 (1.0-1.4)^*^ 147	203	
Sit-and-reach (cm)^3^ x̄ (SD)	27.0 (6.4)	24.6 (6.5)		25.4 (6.9)	22.7 (6.8)	−10.6	25.5 (7.1)	23.6 (6.6)	−7.5
β (95% CI)^2^ *n*	**−2.3 (−3.4;** −1.2)^***^ 365	205	–8.9	**−2.8 (−4.0;** −1.6)^***^ 459	298		−2.3 (−4.1;−0.5)^*^ 153	204	
Plate-tapping (s)^1^ x̄ (SD)	15.6 (1.9)	14.6 (1.9)	–6.4	14.8 (1.8)	14.0 (2.0)	–5.4	14.1 (1.9)	13.4(1.8)	−5.0
β (95% CI)^2^ *n*	**−1.0 (−1.4;** −0.6)^***^ 367	205		**−0.7 (−1.1;** −0.3)^**^ 59	298		**−0.6 (-1.1;** −0.2)^*^ 153	204	

1 A lower value indicates a better test score

2 2015/2017 vs. 2006

3 A higher value indicates a better test score

4 Data not normally distributed, therefore the median (x̄) and interquartile range (IQR) (25^th^-75^th^ percentiles) are provided.

5 Data have been log-transformed, therefore the β should be interpreted as a ratio. Bold is significant, by

* p < 0.05,

** p < 0.01,

*** *p < 0.001*.

11- and 12-year-old boys scored significantly worse on “leg-lifting” in 2015/2017 vs. 2006 (β of log-transformed data = 1.1 and 1.2 s). In girls, no significant differences were found.

## Discussion

This study investigated differences in neuromotor fitness in Dutch 10–12-year-old children in 2006 vs. 2015/2017. Children scored worse in most age and gender categories in 2015/2017 on speed and agility (“10 × 5 meter run”) and flexibility (“sit-and-reach”). 10-year olds scores worse on upper body strength (“bent-arm hang”) and 11-year-olds on explosive leg strength (“standing high-jump”). In contrast, children in 2015/2017 performed better on coordination/upper limb speed (“plate-tapping”) vs. 2006.

In contrast to a Dutch study comparing neuromotor fitness scores of children between 2006 and 1980 (10), the current study found no significant differences on trunk and leg strength (“leg-lifting”) and improved scores on coordination and upper limb speed (“plate-tapping”) in 2015/2017 vs. 2006 in most age and gender groups. A possible explanation for the improved “plate-tapping” scores could be the increase in computer use and gaming (25). Playing certain video games can promote fine motor skills and movement coordination (26), which may have contributed to children's improved “plate-tapping” scores. The item “leg-lifting” showed only worse scores in 11- and 12-year-old boys. In 2006 vs. 1980 all age and gender categories scored significantly worse on upper body strength (“bent-arm hang”), while in the current study significant worse scores were only found in 10-year olds in 2015/2017 vs. 2006. Lastly, in 2006 vs. 1980 children in all age and gender categories scored significantly better on explosive leg strength (“standing high-jump”), while the current study only found significantly worse scores in 11-year-olds.

Previous studies evaluating motor fitness of children over a longer time period showed mixed results. Several studies in Europe and Canada observed worse scores on most items of the motor fitness tests over the past two decades (11, 12, 27). In contrast, one study in Portuguese children demonstrated better scores on speed, trunk strength, and flexibility in 2013 compared to 1993, and no changes in explosive leg strength (28). The authors contributed the improvements to more access to and participation in organized sports of Portuguese children over this time period. The Dutch study comparing neuromotor fitness scores of children between 2006 and 1980, found worse neuromotor fitness scores in 2006 on upper body strength, flexibility, speed and agility, trunk and leg strength, and coordination and upper limb speed (10). The current study again shows significantly worse scores in 2015/2017 vs. 2006 on speed and agility, and flexibility in boys aged 10–12 years and girls aged 10 and 11 years. The declines on these test-items were small, for example only about 1 s on the “10 × 5 meter run,” but differences are comparable to and in some instances larger than reported in the study comparing scores from 2006 to 1980 in the Netherlands (10). Our finding that the downward trend in certain neuromotor fitness scores from 1980 to 2006 has continued from 2006 to 2015/2017, may have important implications for physical activity enjoyment and participation and as a result future health (29).

The downward trend over the last decades in children's neuromotor fitness may be related to downward trends in children's motor competence (30). A study funded by the Dutch ministry showed lower levels of motor competence (balancing, swinging on a rope, aiming at a high target, catching and throwing a small ball via the wall and playing tennis against a wall) in Dutch children from 2006 vs. 2016 (31). The Dutch government responded to these alarming results by implementing several policies to improve children's motor competence (32), such as the recent adoption of an amendment that obligates Dutch primary schools to provide at least 2 h of physical education per week (33). In the past decade a number of Dutch policies have focused on improving physical activity and/or motor competence of children. Practical implications of our results are that upper-body strength and flexibility need specific attention in physical education or physical activity interventions. Implementing internationally standardized tests as part of the school curriculum could provide more insight in time trends and risk groups, and consequently lead to better tailored interventions. Future research has to determine the effects of implemented policies on children's neuromotor fitness and motor competence, as well as physical activity levels and health indicators.

A major strength of this study is the large sample size, including 1986 children in the 2006 sample and 1,474 in the 2015/2017 sample. The use of the same fitness test items and a standardized measurement protocol further strengthens this study as non-standardization of tests has been a problem in previous studies (11). Lastly, the current study compares neuromotor fitness levels in children in 2015/2017 vs. 2006 and such recent data was not available yet. A major limitation of this study is that no data was available at both the individual and school level on potential covariates such as physical activity level, socioeconomic position, cultural background, and BMI, while these could have influenced the results. The schools neighborhoods for example differed in the percentage of people with a non-Western background, with ~11.4% in 2006 and 23.1% in 2015/2017 (34). We adjusted for the degree of urbanization of the school's neighborhood, as that was the only data available. Unfortunately, we could also not adjust for BMI. A previous study including the 2006 MOPER data showed that even when children with overweight and obesity were excluded from the analyses, most declines in children's neuromotor fitness scores from 1980 to 2006 remained significant (10). This suggests that the inferior neuromotor fitness scores found in current generations are not only due to the higher prevalence of overweight, however, we could not verify this in the current study.

## Conclusions

The current study found that the previously observed downward trend in most components of neuromotor fitness among 10–12-year old Dutch children from 1980 to 2006 continued from 2006 to 2015/2017. Persistent low scores were found on most strength components and worsened scores on speed and agility and flexibility (with the exception of 12-year-old girls for the latter item) in 2015/2017 vs. 2006. This downward trend in neuromotor fitness can have important implications on physical activity enjoyment and participation and thereby future health. Therefore, improving children's neuromotor fitness from an early age should be a larger public health priority and be reflected as such in local and national policy.

## Data Availability Statement

The raw data supporting the conclusions of this article will be made available by the authors for scientific research.

## Ethics Statement

The iPlay (2006.129) and Kids in Action (2016.366) study were approved by the Medical Ethics Committee of the VU Medical Center. Written informed consent for participation was not provided by the participants' legal guardians/next of kin because: in 2015, the MOPER test was used as a student monitoring system in school and therefore informed consent was not obtained; in 2006 and 2017, parents of eligible children received a letter containing information on the study and a form to decline their child's participation.

## Author Contributions

TA, MC, and MA designed the 2017 study. MA coordinated and led data collection in 2017 with AB assisting the data collection. DC and MC designed the 2006 study with DC coordinating and leading data collection. AB conducted the data cleaning and assisted in the first draft of this manuscript. MA wrote the manuscript with DC, MC, and TA providing critical input and feedback. JT assisted with the data analysis. All authors contributed to the article and approved the submitted version.

## Conflict of Interest

The authors declare that the research was conducted in the absence of any commercial or financial relationships that could be construed as a potential conflict of interest. The handling editor declared a past collaboration with several of the authors MC, TA.
